# A qualitative study examining the presence and consequences of moral framings in patients’ and mental health workers’ experiences of community treatment orders

**DOI:** 10.1186/s12888-015-0653-0

**Published:** 2015-11-06

**Authors:** Sharon Lawn, Toni Delany, Mariastella Pulvirenti, Ann Smith, John McMillan

**Affiliations:** 1 Flinders Human Behaviour and Health Research Unit, Department of Psychiatry, Flinders University, Flinders Drive, Adelaide, 5042 Australia; 2 Southgate Institute for Health, Society and Equity, Flinders University, Flinders Drive, Adelaide, 5042 Australia; 3 Discipline of Public Health, Flinders University, Flinders Drive, Adelaide, 5042 Australia; 4 CARE Inc, Adelaide, 5162 Australia; 5 The Bioethics Centre, University of Otago, Frederick Street, Dunedin, 9016 New Zealand

**Keywords:** Community mental health, Community treatment orders, Coercion, Empathy, Moral Framing

## Abstract

**Background:**

Mental health recovery involves acknowledging the importance of building the person’s capacity for agency. This might be particularly important for patients on community treatment orders (CTOs - which involve enforced treatment for their mental illness), given limited international evidence for their effectiveness and underlying concerns about the use of coercion by workers and systems of care towards this population of people with mental illness.

**Methods:**

This study sought to understand how the meaning of CTOs is constructed and experienced, from the perspective of patients on CTOs and workers directly administering CTOs. Qualitative interviews were conducted with South Australian community mental health patients (n = 8) and mental health workers (n = 10) in 2013–14. During thematic analysis of data, assisted by NVIVO software, the researchers were struck by the language used by both groups of participants and so undertook an examination of the moral framings apparent within the data.

**Results:**

Moral framing was apparent in participants’ constructions and evaluations of the CTO experience as positive, negative or justifiable. Most patient participants appeared to use moral framing to: try to understand why they were placed on a CTO; make sense of the experience of being on a CTO; and convey the lessons they have learnt. Worker participants appeared to use moral framing to justify the imposition of care. Empathy was part of this, as was patients’ positive right to services and treatment, which they believed would only occur for these patients via a CTO. Workers positioned themselves as trying to put themselves in the patients’ shoes as a way of acting virtuously towards them, softening the coercive stick approach. Four themes were identified: explicit moral framing; best interests of the patient; lessons learned by the patient; and, empathy.

**Conclusions:**

Experiences of CTOs are multi-layered, and depend critically upon empathy and reflection on the relationship between what is done and how it is done. This includes explicit examination of the moral framing present in everyday interactions between mental health workers and their patients in order to overcome the paradox of the moral grey zone between caring and controlling. It suggests a need for workers to receive ongoing empathy training.

## Background

The importance of mental health services adopting a recovery orientation is embedded into many Mental Health Acts and policy statements [1, 2]. A recovery orientation requires that ‘the patient’ be treated as a moral agent who is active in their own recovery process. Central to recovery-based practice is that people with mental illness are able to exercise rights and experience membership of a community [3]. Working toward recovery involves acknowledging the person’s capacity for agency; how they are enabled to maximize a positive sense of self as a citizen, and minimize threats to agency by what Fisher [4] describes as ‘being done to’ within systems of care (p.12). Anthony [5] defines recovery as, “a deeply personal, unique process…a way of living a satisfying, hopeful and contributing life even with limitations caused by the illness” (p.13). Anthony’s definition is important, “because he emphasizes that recovery is a personal journey where people reconfigure and reconstruct their lives. This naturally leads to giving thought to the kind of environments which facilitate the journey, and in which positive rights are maximized”(p.290) [6].

One group for whom a recovery orientation might be particularly important is those who are subject to a community treatment order (CTO). In South Australia a CTO application is made under the Mental Health Act, usually by a specifically authorised Tribunal, on application from a medical practitioner, a mental health clinician, a guardian, medical agent, relative or other person connected to the person. A CTO requires a person with a mental illness to comply with treatment for that mental illness even if they do not want to. If a person on a CTO refuses treatment, the treatment provider can authorise enforced treatment. This may involve the person being brought to a treatment facility by police.

Similar requirements exist in other jurisdictions. Criteria for CTOs vary; however they share some common features:

- The person has a mental illness:
- Because of the mental illness, the person requires treatment for the person’s own protection or for the protection of others from harm; and
- There is no less restrictive option for ensuring appropriate treatment of the person’s mental illness.

CTOs make mental health treatment mandatory and that fact alone creates a tension with a recovery-oriented approach. Rates of CTO use in Australia range from 30.2 per 100,000 people in Tasmania, to 98.8 per 100,000 in Victoria [7]. It seems unlikely that these differences are due to jurisdictions having greater or lesser numbers of people requiring involuntary community treatment, but involve other factors such as the ease with which CTOs are created or varying therapeutic responses among service providers. High and variable rates of CTO use are of concern because this, “raises questions of whether the measure is being appropriately targeted to a high needs groups, or whether it has become a default option in defensively-oriented mental health services” (p.355) [8].

Fundamental to most Mental Health Acts in Australia, and many other countries, is the idea that people have the right to be treated in the least restrictive environment. While CTOs appear to promote this ideal because they involve people being treated in their community, research has shown that many people placed on a CTO experience negative feelings about their involuntary treatment, and that it exacerbates their feelings of stigma and disempowerment [9–13]. Gault et al. [14] describe the patient who is subject to CTO legislation as ‘a discredited identity’ who, in their attempts to regain some control, resorts to ‘playing the game’ of appearing to be compliant because appearing to behave in a certain manner yields positive results. Clarke [15] describes patients as, “objects of intensified surveillance, criminalization and incarceration” (p.458). Chow and Priebe’s [16] examination of psychiatric care and institutionalization highlight the need for more research on how patients’ adapt their behavior to care.

A number of concerns have been raised about the use of CTOs. These focus upon the impact of CTOs on civil liberties [17, 18]; that they are instruments of social control and represent, “a strategy to spend less than the best care would cost” (p.473) [19]; and that assessment of risk of future harm to self or others on which CTOs are based is unreliable and based upon weak evidence that they reduce risk [20–22]. Sawyer [23] argues that the growing focus on risk has, “diminished the significance and legitimacy of therapeutic responses” (p.287). She describes mental health service providers as, ‘psychiatric risk managers’ in communities focused on containment and ensuring security. Dunn et al. [24] examine these processes in details, in particular, making threats and offers to patients as strategies to increase treatment adherence. Chow and Priebe [16] argue that a process of ‘re-institutionalization’ has occurred in several countries since 1990, and that this is clearly evident in inpatient care but also in the community.

Mental health service patients and workers are placed in a complex and contradictory paradox in which caring whilst simultaneously policing becomes complicated. This is because CTOs may, in fact, deter people from seeking help and engaging, hinder medication adherence [25], and reinforce negative stereotypes about people with mental illness as dangerous [26–28]. Callaghan and Ryan [20] state that, “such schemes will arguably lead to the denial of treatment for patients in genuine need and to forced detention and treatment of patients whose refusals should perhaps have been respected” (p.613) in the same way as any other patient with capacity. Capacity tests are not integral to Australian Mental Health Acts and patients deemed at risk of harm to themselves or others can be treated without consent if they have a mental illness, whether they have decision-making capacity or not; though a number of Australian states are reviewing their Acts [29, 30].

Finally, there is little evidence for CTOs producing positive clinical outcomes [31]. Most research has shown that CTOs do not reduce readmission rates or duration, or increase time to readmission or treatment adherence [17, 32–34]. A study of 90 patients by Suetani et al. [35] found a trend for greater compliance to intra-muscular depot medication by those not on a CTO compared with those who were. They concluded that CTOs become a double-edged sword because, while forced cooperation might lead some patients to accept medication in the long-term, other patients might associate the experience with the loss of autonomy and develop negative attitudes to medication, help-seeking from mental health services and indeed the medical profession, more generally.

Newton-Howes and Banks [36] undertook statistical modelling to predict which CTO patients might experience them as either positive or negative to their care. They found that, while many patients described greater coercion when subject to a CTO, many patients felt they were better off when their mental health was managed with a CTO; suggesting a need for further research. The conflicting view of CTOs as coercive or beneficial, “does not describe a simple dichotomy between paternalism and autonomy; but an experience characterized by intense practice, moral, existential and legal complexity and uncertainty” (p.350) [37]; this tension exists, “in the moral grey zone between caring and controlling” (p.50) [38]. Our study seeks to address the need for a more rich and nuanced description (from the perspective of both mental health workers and patients) of the experience of coercion for people who are subject to CTOs, given this complexity. Understanding how the meaning of CTOs is constructed requires paying attention to first person lived experience accounts; creating significance and ‘moral meaning’ from that experience [39]. To explore these experiences within the data, we used a moral framing approach.

## Methods

### Moral framing

This paper reports on the moral framing that emerged from the data collected for a broader study of the experience of CTOs from patients’ and workers’ perspectives. The research question guiding the broader study was: What are the experiences of people with mental illness who have been placed on Community Treatment Orders (CTOs) and to what extent are they supported to overcome the need for an order, as part of that treatment and care period?

The objectives of the broader study were:

1. To understand the person’s experience of CTOs and any perceived barriers and facilitators to their participation in their own treatment and care planning whilst on a CTO.
2. To identify the perceived barriers and facilitators for great participation in treatment and care for people on CTOs from the perspective of mental health professionals.

During data analysis for the broader study, the researchers were struck by the language used by both groups of participants and so decided to undertake an examination of the moral language apparent in the data, more formally.

Jones [40] defines framing as, “a central organizing idea, or frame, for making sense of relevant events” (p.5). He argues that, “The linguistic perspective is especially well-suited to studying content and construction of frames, as it pays close attention to the structure of language and the process of conveying meaning” (p.6). [40] Jones explains that, “how an issue is framed seems to powerfully effect how the public thinks about and reacts to it” (p.2) [40], especially when an issue is infused with a moral element, accompanied by emotionally charged and moralistic language [41]. Notions of morality are central to healthcare and the therapeutic relationship between workers and their patients [42]. Gray et al. [43] suggest that, “moral action in healthcare involves three elements: the moral agent, the moral action, and the moral patient. A moral agent (defined as an individual capable of intentional action) performs a moral act (an intentional action which affects another either for good or ill) on a moral patient (the object of the moral action that is either deserving of good treatment or at least undeserving of bad treatment)….Deservingness becomes fundamental, and moral framing’s task is to cast certain groups and individuals as either deserving or undeserving of help or harm” (p.4-5) (see also [44]). In this study, the moral agent is the worker, the action is the imposition of a CTO (legal and clinical processes), and the moral patient is the patient subject to a CTO.

### Sample

The sample was drawn from within a South Australian community mental health service serving the needs of approximately 800 people with mental illness (with approximately 10 % of these on a CTO) via a multi-disciplinary case management team of approximately 40 health professionals. Eight patients were interviewed for the study. Patient participants were women (n = 5) and men (n = 3), aged 18 years and over, living in Adelaide, South Australia. All were clients of the State-funded clinical mental health services, currently on a CTO and beyond the first six months of the CTO. Most had experienced being on a CTO several times previous to their current CTO (See Table 1 – All patient participants gave their consent to publish the displayed indirect identifiers within the table). Exclusion criteria were:

**Table 1:** Description of patient participants

	Pseudonym	Gender	Age years	Marital status	Mental Health Diagnosis	Living Situation	Employment Situation ^a^	Number of CTOs	Number of years as client of Mental Health Services	Stage of Current CTO ^b^	CTO On/Off experience ^c^
1	Vicky	Female	Early 50s	Married	Schizophrenia	With husband	Unemployed DSP	2	3	8 months	Intermittent
2	John	Male	Mid 40s	Single	Schizophrenia	Alone (long-stay locked institutional care during his 20s)	Unemployed DSP	15 + (lost count)	28	8 months	Continuous
							Intermittent work				
3	Peter	Male	Early 30s	Single	Schizoaffective Disorder	Alone	Unemployed DSP	6+ (lost count)	14	9 months	Continuous
4	Jessica	Female	Early 50s	Single	Schizophrenia	Alone	Unemployed DSP	5	17	8 months	Intermittent
5	Susan	Female	Mid 30s	Divorced	Schizophrenia, Borderline Personality Disorder, Obsessive compulsive disorder, Depression	Hostel	Unemployed DSP	4	12	6 months	Intermittent & Continuous
6	Thomas	Male	Late 20s	Single	Schizoaffective disorder, Bipolar Affective Disorder	Alone	Unemployed DSP	6 (lost count)	9	6 months	Intermittent & Continuous
7	Joan	Female	Early 50s	Divorced	Schizophrenia	Alone	Unemployed DSP	4 (cannot remember)	13	9 months	Intermittent & Continuous
							Intermittent work				
8	Jenny	Female	Late 20s	Single	Schizophrenia, Borderline Personality Disorder, Obsessive Compulsive Disorder, Depression	Alone	Unemployed DSP	2	10	6 months	Intermittent

^a^*DSP* Disability Support Pension, government payment

^b^All CTOs were for 12 months

^c^For example, Susan has had intermittent and continuous experiences of being on a CTO. She had her first 12-month order, then had 2–3 years without an order, then has had 3 orders in a row

- Intellectual or cognitive disability that renders the person unable to provide informed consent;
- Current suicidality or other risk as determined by the mental health services;
- Case-note alert signifying two person contact was required.

Workers were drawn from a range of professional disciplines (2 psychiatrists, 3 nurses, 3 occupational therapists and 2 social workers). They were either community treating doctors or case managers. The worker participants were currently employed for 5 years or more, to ensure an established degree of experience and involvement in CTO applications and their administration.

### Recruitment

Participants were recruited via their mental health community case managers who determined their ability to provide informed consent and not pose any risks during the interview. The researchers provided information about the study in a presentation to the community mental health team who were then asked to identify potential patient participants from their caseloads and provide them with an information sheet and consent form. In most cases, the patient participants contacted the lead researcher independently of the case manager to ensure anonymity of their participation. In some cases, the patient participant was happy for the case manager to provide their contact details to the lead researcher. The lead researcher then contacted the patient participant to arrange a time and place to meet to conduct an interview.

Worker participants were recruited via a general global email sent to the service’s clinical lead for distribution to staff.

### Ethics

Permission for the study was sought from the clinical and service directors of the mental health service. Ethics approval was granted by the SA Health Human Research Ethics Committee.

### Data collection

The research was explained, voluntary consent was confirmed and a consent form signed prior to commencement of interviews with all patient and worker participants. For patients, interviews occurred in their home (n = 4), a public location where the patient felt comfortable (n = 2), or the lead researcher’s office (n = 2). All worker interviews (n = 10) occurred in a private office at their service, during their usual working hours, at a time convenient for them. All interviews were audio-recorded with consent, and professionally transcribed to enhance recall and rigor, except for two patients who requested that an audio-recording device not be used. Coincidentally, these two participants also chose to undertake the interview in a public location away from their home. They were happy for the researcher to collect them from their home and return them there after the interview. Extensive notes were taken during the interviews with these participants. Due to the potential to discuss highly sensitive information about their experience of being on a CTO or administering a CTO, participants were offered support to link with existing supports or services (e.g. Case managers for patients or Employee Assistance Program for workers); however, none reported needing this assistance.

An interview guide was developed in consultation with a project reference group, informed by the reviewed literature.

Worker and patient interview guide

- Workers
  1. Describe what you think of CTOs for people with mental illness? Benefits? Concerns?
  2. Describe your own experience of delivering treatment and care to patients on a CTO?
  3. What factors do you consider in determining the level of involvement of the person and their decision-making capacity when applying for a CTO and/or providing treatment and care during the time that they are on a CTO?
  4. Describe your experience of the Guardianship Board hearing process and of applying for a CTO, or providing input to an application to the Board?
  5. What types of support do you provide to patients while they are on a CTO?
  6. Are there circumstances that prevent you from providing the support you would like to provide to patients on a CTO? Explain?
  7. What do you perceive as the impacts for patients of being on a CTO?
    Benefits? Problems? Impacts for you/the service/ others?
  8. Are you involved in the development of mental health care plans for patients on a CTO? If so, your experience of these and processes followed? Patient copy? How often reviewed? Your perceptions of what patients think about them?
  9. How could MH services improve how they provide support to people on a CTO?
  10. Do you have any other comments to make about your experiences of providing treatment and care to people on a CTO?

- Patients
  1. Description of how you came to be on a CTO? How long? Others? Recollections of interactions with mental health staff and Guardianship Board hearing?
  2. Description of your experience of receiving contact with MHS since being on a CTO? Case manager? Psychiatrist? Other support people?
  3. Level of involvement in making or sharing decisions about your treatment since being on a CTO? Examples? How you felt about this?
  4. Level of involvement in making or sharing decision about other parts of your life since being on a CTO? (eg. Psychosocial support needs). Examples? How you felt about this?
  5. What support does the mental health case manager provide to you as part of their contact with you?
  6. What do you perceive as the impacts for you of being on a CTO? Benefits? Problems?
  7. Do you have a mental health care plan? Your view of it? Have you seen it/got a copy? How often is it reviewed with you? Your involvement in its review?
  8. Do you feel that your life has changed since being on a CTO? Why? Why not? If so, what has changed?
  9. How could mental health services improve how they provide support to people on a CTO?
  10. Do you have any other comments to make about your experience of being on a CTO?

The lead researcher (SL), who conducted all interviews to ensure consistency, was a mental health consumer advocate and person with over a decade of experience as a mental health professional. They did not have any current relationship or connection with any participants of this study (see limitations for further detail). Another member of the research team (MP), a researcher without experience as a worker in mental health services, accompanied the lead research for two worker interviews, to provide an independent critical lens on the interview exchange. All participants were provided with the opportunity to view and verify transcribed interview accuracy, to further reflect on their comments. The researchers met routinely to discuss the meaning of the data as interviews proceeded. Where possible, these sessions were audio-recorded to capture the dialogue. Reflective notes were made after each interview to capture the context of the interview and to record the interviewer’s observations.

### Data analysis

**Fig. 1:**
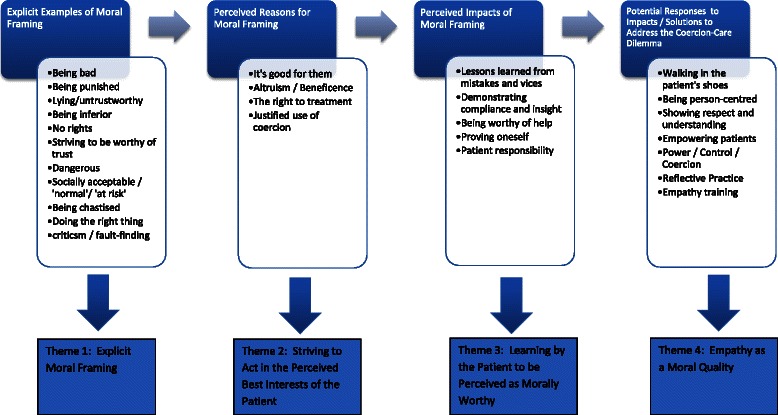
Broad Coding Framework

Initially, the researchers performed open-coding of four randomly chosen interview transcripts (2 patient and 2 worker transcripts), independently of each other. They then met to discuss and debate their assigned codes to establish an agreed coding framework. This framework was used to code all remaining interviews with the assistance of NVIVO 10 software. Following an initial round of open-coding, selective-coding was applied to identify key themes in participants’ discussions. This involved an in-depth constructionist exploration of themes [45], directed by researchers’ identification of the use of moralistic framings and the need to explore this further (see Table 2 for an example of this process). Once approximately three quarters of all interviews were coded in this way, the researchers met again to discuss and determine core and sub-themes. Through this discussion, the research team determined an overarching framework in which to position the themes. This framework identified explicit examples of the use of moral language, participants’ perceived reasons for their views within a moral context, the perceived impact of these views and patient/worker reactions, and participants’ views on what needed to happen to address the situation (See Fig. 1). Once all interviews were coded, the research team met again to finalize the themes. As this was an exploratory study in an area that has not been researched before, we were not aiming for data saturation.

**Table 2:** Examples of data analysis process

Examples of Transcript Text	Open coding	Research Team Discussion	Selective coding
Reference 2		<Internals\\CTO Analysis meeting MJS Feb 2014 coded>	
but this time around I’m staying straight in that – you know beforehand I did it with – we’d get the whole scenario in smoking marijuana, then I’ve went into xhospital, got detoxed all of you know, and this time what I’m doing I’m going through it straight. Because I’ve understood what I did it then and that	Changed thinking about actions	Reference 1	
	Now sees things differently	The last sentence there, where he says they grabbed my folder off the shelf and started looking at it because hey [John’s] toeing the line, that he wants to get better, he knows what not to do.	Being compliant
Reference 3	Now listens to MHS providers	Reference 2	Learning his lesson (Theme)
This time around now that you guys have – I believe in you guys and that because I didn’t understand it beforehand, and this time around you know straight you know, even alcohol I might just have a couple of beers a week.	Becoming more well	So this is kind of a deep little metaphor about orientating oneself towards seeing that there’s an issue and addressing it.	Trust in workers’ view
	Workers ready to work with him	Reference 3	
Reference 4	Proving he can change	Yeah, he’s very compliant; he’s very passive to the service. It’s, I’ll do what they say –	Patient showing potential /being good, so workers respond
I was waking up out of it and that and I showed signs that I was on a new road for Clozapine. So then eventually you know that admission, they grabbed my folder off the shelf and started looking at it because ‘hey John’s toeing the line and that, he wants to get better, he knows what not to do and everything, he’s showing signs of coming down to the problem and we’ll start helping him.’	Learned from bad experience	Well yeah, he set up here, again on the top of page three after he talks about the hospital being a wake-up call and reality check he says “don’t do this again because it’s not worth it, stay on the medication then you won’t have to be subject to the pain to get back to where you were before you were admitted” so he’s got – now he’s got a sense, as he articulates there, he’s got a sense of how painful it was and what a struggle it must have been for him to go through that process but that’s what he’s saying now.	Patient now worthy of helping(Theme)
	Patient wanted to get well		Learning lessons (Theme)
	Compliant		Worthy/ honest (Theme)
Reference 5	Trying hard to be well		Worthy of helping because promises to be compliant (Theme)
now I can see that wasn’t the right way to go and that, you know I done it, I seen it, I paid for it			
Reference 6			
I was trying so much to get myself better			
Reference 7			
he wants to get better, he knows what not to do and everything			
References 8			
I was always loyal to take my lollies you know every night or morning and that sort of thing, when I was - you know and I learnt to sort of religiously take them unless I got unwell for some sort of reason.

## Results

Moral framing was apparent in participants’ constructions and evaluations of the CTO experience as positive, negative or justifiable. Some participants made constructed narratives that referred to ‘having good morals’, ‘being morally good’, ‘being morally worthy’, ‘behaving well’, ‘behaving morally or honourably’, or ‘being good enough’. Negative moral framing referred to ‘being sinful or lacking morals’, ‘failing’, ‘being weak’, ‘having faults’, ‘being imperfect’, or ‘being wicked’. Most patient participants appeared to use moral framing to: try to understand why they were placed on a CTO; make sense of the experience of being on a CTO; and convey the lessons they have learnt. Worker participants appeared to use moral framing to justify the imposition of care. Empathy was part of this, as was patients’ positive right to services and treatment, which they believed would only occur for these patients via a CTO. Workers positioned themselves as trying to put themselves in the patients’ shoes as a way of acting virtuously towards them, softening the coercive stick approach which may be considered otherwise unethical and too harsh. Four themes are discussed: explicit moral framing; striving to act in the perceived best interests of the patient; learning by the patient to be perceived as morally worthy; and, empathy as a moral quality (see Fig. 1). Pseudonyms are used for all participants.

1. Explicit Moral Framing

Patients used explicit moral language to describe themselves and others like them who were on CTOs. Their descriptions frequently involved them seeing themselves as deviating from a norm of some kind and needing to have their faults corrected in some way by others who were seen as more worthy and having more authority to know what the ‘right’ course of action was to take. Joan, a lady with schizophrenia in her 50s, had been a client of mental health services for many years and had been on a CTO for much of that time due to a perceived lack of insight and being non-compliant with medications when not on a CTO.

*(Joan) I really believe CTOs are for people that have done something wrong and need to be looked after, yeah.*

John, a man with schizophrenia in his 40s, who had been a client of mental health services for most of his adult life and had been subject to a CTO many time before, described his perceptions of being in hospital and on a CTO in great detail, as ‘for my own good’. He stated that workers had the right to detain and restrain people with mental illness, if needed. During the interview, John’s manner was very apologetic and passive, as if he had taken on the belief that he was being punished somehow.

*(John) This time what I'm doing I'm going through it straight. Because I've understood what I did it then and that…now I can see that wasn't the right way to go and that, you know I done it, I seen it, I paid for it…I was trying so much to get myself better…I was always loyal to take my lollies [tablets] you know every night or morning… and I learnt to sort of religiously take them unless I got unwell for some sort of reason.*

Vicky, a lady with schizophrenia in her 50s, explained how she had come to be on a CTO following a hospital admission during which she was receiving care from a doctor who was not previously known to her. The CTO was imposed as a result of her lying about taking her tablets to the hospital treating team during the early part of her admission. As Vicky’s health settled, she admitted to workers that she had lied. Vicky’s description of what happened next highlighted her perception that the lying had been constructed by workers as a moral transgression that set her up as a certain kind of person who could not be trusted and who needed to be punished. The belief was that she would now go down a particular road, to being a person who lies, a difficult person who cannot be trusted, and therefore needed a CTO to ensure that she took her medication from now on, despite her honesty with staff.

*(Vicky) See I should have gone back to my original psychiatrist and he should have said ‘Well all right, you lied, so what?’ and I thought that's absolutely fair. He should have said ‘Well you lied. So what? Let's start again’… It didn't seem to be a choice to me. It was about everybody agreeing to me being on the Risperidone rather than me having a choice and saying no, I would rather go back to my psychiatrist I'm dealing with…I didn't get a second chance…they then used the threat of an order…’Since you won't take your tablets, we're not going to trust you again, we want you to have an order’… How do you engage with people therapeutically even when you're taking away their rights?*

Jenny was a young woman with schizophrenia in her 20s who had been on a CTO intermittently since her mid-teens. She felt particularly negative about her experience of being on a CTO because, despite being an articulate and intelligent person, these qualities were not enough to have her views heard. Like Joan, Jessica and Peter, Jenny’s home was immaculately decorated with cherished objects which she took pride in showing to the interviewer. They signified a ‘normal’ life beyond mental illness; a haven in which she spent much of her time and which, in the interviewer’s clear perception, few others entered or were allowed to enter. She described her experience of the CTO hearing in a way that showed, explicitly, that she felt she was viewed by others around her as a morally lesser person, without the same standing as a citizen.

*(Jenny) “You have no rights anymore, you can be discriminated against…They think you’re making it up or - like, because obviously you’ve got a mental illness so everything you say is completely invalidated and discredited… [Of the CTO hearing] …the lady who conducted the hearing commented, she thought that I was articulate and intelligent, but it didn’t really help me very much.”*

In contrast, Jenny’s experience of being involved in this study was different because she felt listened to and not judged.

*(Jenny)[Of the interviewer] You respect my autonomy and my right to decide for myself, you know; who I want in my home…and then when you have people, like, police and stuff violating that stuff…because there’s such hatred for people with mental illness in the community….* They suddenly start being nasty to you, and it’s like: ‘What did I do wrong?

Worker participants also used explicit moral language to describe the tensions involved in imposing a CTO as part of their role with patients. They seemed to both recognise and resist their role in actively constructing the boundaries of moral transgression, which separated right from wrong and which became the basis for workers expressing the right to detain over the patients’ right to choose. Workers like Kim were clear that they wanted to be seen as virtuous towards patients. Kim’s assumption is that the patient wants to feel better; however, it is unclear whether she asks them this directly, which seems to be a moral question itself.

*(Kim) I hate that word power, but they talk about 'powers of detention', which is a horrible …I've heard people talk about, "But I haven't done anything wrong", seeing it as a bad thing or a punishment, and I like to think of it as more of a safety net to ensure they have some support or contact…Look, I like to think that I treat all clients, similar, as people, not just consumers. I like to think that I'm there, I'm interested in them. I want them to feel better. Yes, some of them are so disempowered. They've often been institutionalised, they've kind of – they are like, ‘I haven't got really any rights.’*

The explicit moral language used by Kim and Robyn, of viewing patients as having positive potential and existing within a system in which they are striving to be worthy of trust and autonomy, was evident in how they described the dilemmas and challenges they faced when working with patients on a CTO.

*(Robyn) When does a person get a chance to prove that they can do this on their own? That stuff upsets me because I see it as a total removal of your rights.*

*(Kim) And that's what I said to him, "Through that hearing is the last thing we wanted to do was to come with police and everything" but we were worried because he wasn't eating, he wasn't answering his door, he was walking in the heat, there was just all this type of stuff, so it was…And particularly people who, when I first started out, there was a lot of potential for them to do well.*

Robyn went further to describe problems with people with mental illness being perceived as ‘untouchable’ and dangerous:

*(Robyn) I’m really conscious in mental health, we did get taught if somebody’s psychotic you don’t touch them…and that bugged me for a long time because…for me it was a natural thing, is that caring, nurturing…so what I do now is actually offer my hand to shake the client’s hand…because, for a lot of people, they don’t actually receive that touch. Nobody touches them, and I just think, ‘How do people survive?’… [Recounting working with a patient in the acute locked ward] I said, ‘Oh, it’s meal time’ and he said, ‘Will you come and pray with me first?’ I’m like, ‘Okay, sure’…and someone [another worker] comes along and says, ‘What are you doing?’ I said, ‘He’s praying’, ‘Well, you shouldn’t be in here on your own.’*

Tim reflected on the nuanced nature of service providers’ and the Guardianship Board’s processes in determining whether patients’ behaviour was seen as legitimate and socially acceptable, or not. He questioned what should be seen as ‘normal’ versus ‘at risk’ or ‘dangerous’ behaviour, as part of determining whether a person required compulsory treatment for their mental illness.

*(Tim) I think one of the proper intentions of a review body like the Guardianship Board is trying to pin clinicians down to being precise when they talk about risk, of course, because the Guardianship Board absolutely appals dialogue, they attempt to compel people to behave in certain ways, but these are proper questions. When you’re saying somebody presents a risk to others, what do you mean? Do they shout around in their backyard at 3 am or do they throw their rubbish over the fence?…And all of those are perfectly legitimate reasons to encourage somebody towards treatment I think; but, the language gets used carelessly and dangerousness can mean a whole bunch of things…There is somebody distancing themselves from their social supports, there is somebody inviting retaliation because they wander around pushing strangers, there is somebody tinkering around with the fuse box because they think somebody is bugging their house and these are legitimate ways in which people can expose themselves to danger.*

Tim described how some of his colleagues take on a moral interpretation when patients resist taking medication.

*(Tim) Sometime, I think some key workers think of people stopping their treatment that there’s a wilful element to it…[On workers’ response to such patients] I’d go as far as almost in some way punished or chastised for not taking their medication.*

In a later part of the interview, Tim described the role of the system of care on shaping patients’ behaviours and workers’ behaviours towards patients, and pressure to ‘do the right thing’. His remarks below suggest that, because CTOs are a justified way in which to treat some patients, they gain a kind of moral inertia and become the routine way in which patients are treated.

*(Interviewer) Some of the people we’ve interviewed said that, ‘The only way that I would take medication is if I had an Order. If there’s no Order then I’m not taking it’.*

*(Tim) Well, yes, yeah, and that’s sort of, almost an institutional response. We’ve trained people that, ‘You will only stay in hospital if you’re on an ITO [interim treatment order], you’ll only have your medication if you’re on a CTO’ rather than finding any other point of common ground to negotiate these things…Mental health clinicians get habituated to depriving people of their liberty because we are so convinced that we are doing the right thing, and we have a sense of having a finite number of tools with which to do the right thing by people, or you just don’t think about what it means to have your choices taken away, and I think people chaff under that…if effective treatment really can’t be* delivered in non-coercive ways then there are people who absolutely deserve a trial of treatment [as part of a CTO].

Tim’s further comments suggest that a negative moral frame was pervasive within mental health service culture, beyond workers’ interactions with patients; that it was also inherent within their professional interactions with peers.

*(Tim) The offer of clinical supervision is seen as having a punitive agenda. It’s seen as implying criticism and judgment and fault finding.*

(2) Striving to Act in the Perceived Best Interests of the Patient

Similarly to patients, workers expressed taking actions and holding views about CTOs that were for the good of the patient. As Kim stated, *“Ultimately, you want the best for the client.”* She went on to talk about a particular male patient’s situation; using emotive language to describe the moral dilemmas she faced as part of supporting the doctor applying to put him on a CTO.

*(Kim) He was saying, ‘Thank you. I know you're trying to help’. He kept saying that so we felt really awful…he was a risk to himself and we just actually had to get him to hospital that day…And you certainly care, you really want to do the best for that client to get good outcomes, and you know that they're often not in agreeance with it, and it is tricky…[of the CTO hearing] So I guess it's sort of being careful not – you know, offering to talk and be there but then also knowing that you're going to be for the hearing so you're going to be there presenting; that, ‘Actually, I think this is good and this will be in your best interests’, but –‘*

Several workers expressed the need for CTOs as a positive right for their patients and themselves as service providers; that the use of coercion via a CTO was justified because it ensured some patients received access to mental health treatment and care, as exemplified by Judith.

*(Judith) Well, my opinion is that, to be afraid to use the law and to not be treating these people in the best way that you can, is really doing them a massive disservice. They have the right to be treated and we have the right to treat them.*

The language used to describe the best interests of the patient also extended to how much and if they were involved in decision-making when they were put on a CTO.

*(Vicky, when asked about her involvement in the decision to be put on a CTO) I wasn’t really, no, it was…the doctor made the decision that it was the best thing for me… …I couldn’t debate the fact that whether I had a choice or whether I wanted to – there was already a decision made that I was to be on this Order…but I don’t know how the lady at the end that was there thought it was the best thing for me…it wasn’t like I had any support in that circumstance.”*

(3) Learning by the Patient to Be Perceived as Morally Worthy

Patients described needing to overcome a ‘vice’, or change an ‘unacceptable behaviour’ to get off the CTO and/or to get ‘better’. They appeared to develop the understanding that they needed to emphasize or display positive learning to people in positions of power, to demonstrate that they and their behaviours have improved enough to not need the CTO anymore (i.e. that they are becoming good enough now, learning how they need to behave, learning from their mistakes and past vices).

*(Joan) I learnt to deal with my emotions by not crying so much and not being suicidal.*

*(Susan) It ended up, after two years I was all right. I agreed that I needed to be on one [CTO].*

*(John -* who now sensed how painful his struggle was to go through mental illness relapse*) You come in the hospital and it's a wakeup call…it gives you a bit of a reality check. You say to yourself, ‘Don't do this again because it's not worth it, stay on the medication then you won't have to be subject to the pain to get back to where you were before you were admitted’…But this time around I'm staying straight in that – you know, beforehand I did it with – we'd get the whole scenario in smoking marijuana, then I've went into hospital, got detoxed all of, you know, and this time what I'm doing I'm going through it straight. Because I've understood what I did it, then and that…Now I can see that wasn't the right way to go…I done it, I seen it, I paid for it, but this time around now that you guys have – I believe in you guys and that because I didn't understand it beforehand, and this time around you know, straight you know.*

John’s description of the lesson he learned was almost existential. He described himself as sitting on ‘a shelf’ for a long time and eventually, services seeing him as worthy of being helped, of being saved.

*(John) I was waking up out of it and that and I showed signs that I was on a new road for Clozapine. So then eventually, you know ,that admission, they grabbed my folder off the shelf and started looking at it because ‘Hey, John's toeing the line and that, he wants to get better, he knows what not to do and everything, he's showing signs of coming down to the problem and we'll start helping him’.*

Roxanne described seeing CTOs as a process of learning that the patient was required to go through, that the process was a frustrating one; and implying that, ultimately, the patient was responsible for learning the lessons needed to get off of the CTO, to take control of their life. This is interesting given that the need for a CTO is determined, in part, by an assessment that the person lacks insight and capacity for making decisions that do not involve risks to self or others. Yet, as Roxanne paradoxically observes, recovery often involves feeling able to retake control over one’s life:

*(Roxanne) And I guess that’s the question isn’t it, what have we learnt from this? You’ve been on three CTOs now, what are we learning from this [laughing].*

Thomas, a man in his 20s with schizoaffective disorder who spent much of his time reading philosophy and staying home, portrayed a deep sense of needing to learn, to think his way to solutions to his situation, to be seen to be saying ‘the right thing’. This guise or mask was perceived by the interviewer as an extension of him needing to demonstrate wellness, obedience and goodness to people in power; to demonstrate that he had learned his lesson; that he was an articulate, well read, reflexive person; no longer ‘morally’ lacking; a worthy citizen with potential now. He and the interviewer seemed to be ‘playing this dance’.

*(Thomas) Yeah, I’m paying special attention to the process that I’m going through on a daily basis now because…it’s teaching me that a drug is something that’s a very fine line between pleasure and pain and that goes for all drugs, even the ones that are prescribed to us by psychiatrists, and for that, that’s been a very special lesson for me to learn because my problem and my problems have probably exacerbated the – the initial onset of my mental illness was the fact that I’ve used illicit substances and developed addictive tendencies towards them…Yeah, and my integrity is really on the line because this time, now that I’m equipped with this knowledge that it’s also, like, it’s helping me stabilise my mood and it’s helping dopamine receptors do what they need to do….I can’t prove to my Care Coordinator that I’m ready to adhere to self-management unless I stay off of my drug of choice which is Marijuana and let the psychiatric medication do what it’s meant to do…Yeah, I mean, it’s easier for me to fall into a trap and think that I need to get off the medication as soon as possible but that would be complete hypocrisy on my behalf because I’m not actually, because I’m actually only four days clean off of my drug of choice today, but I had – it might sound a bit like a cliché – but I’ve had the epiphany in recent days…This time I’m reaching out to seek truth in things whether it be Wikipedia and the different pharmaceutical drugs and their treatments, and notions about recovery and studies and stuff like that…Yeah, it’s just like getting over that ego and thinking, ‘Well, I’m not the doctor and if the doctor wants to talk small talk the doctor can talk small talk. If the doctor wants to talk about more deeper stuff, then the doctor gets to talk about more deeper stuff.’*

Jenny described in great detail, how she learned to behave in order to have staff listen to her during an admission to hospital, where she was detained in a locked ward. She had learned that presenting oneself in specific ways was more likely to allow you to be trusted and listened to; whereas, through transgressing the boundaries of acceptable behavior, you become positioned as erratic and essentially unworthy of trust and/or attention. Jenny’s description of her fears makes sense and her way of mitigating the risk to herself in rational, demonstrates insight and shows how morality is constructed and shapes experience.

*(Jenny – describing her concern about her safety in the ward where she perceived herself at risk from a male patient) Yeah, well they actually did believe me not because I came across as rushed but because I was very careful, even though I was in - I had a side effect…it was like a panic attack really bad, but it was a chemically induced panic attack type thing…and I was very careful to present myself in a way that was, like, calm and rational and not to seem like I was angry or upset or - I was too panicked to be upset, to be angry even. I was so scared.*

*(Interviewer) So you’d realised that you had to behave in a certain way in order for them to listen to you or to believe you?*

*(Jenny) Yes, because, when you’ve got the diagnosis, that just overtakes everything. You have no rights anymore, you can be discriminated against…I was terrified of this man next door because I thought, you know, like - he just seemed like scary to me and I was worried about being raped to be honest in the psych ward because I think if it happens, who’s going to believe me, you know. Like, she [the nurse] was threatening; they were threatening to call the code black [emergency response usually involving seclusion and restraint] because I wouldn’t take a drug, because I wanted to be awake at night in case he tried to come into my room.*

(4) Empathy as a Moral Quality

Empathy has been identified as a key skill for adopting a recovery-orientation to mental health care [46]. There are many definitions of what empathy is and conflicting accounts of whether it is primarily a cognitive or an emotional skill [47], but at its core it is the ability to place oneself into the position of another person and thereby understand how things are for them. The ability to put themselves in the patients’ shoes and actually feel what it must be like to be on a CTO was an ability that some workers had and some, perhaps, did not. Their descriptions demonstrate the complexity of working with people on CTOs, how they attempted to express empathy and their perceived understanding of the impacts of being on a CTO, but also the impacts of patients not receiving adequate treatment without a CTO. This empathy was underpinned by a desire to act in morally positive ways towards patients.

*(Kim) I think it's just sometimes having a presence and showing consumers that I'm there and, you know, may not have a relationship with me…”You can come and meet with me, or I can come to you, we can work something if you want to". Some people never, ever, want medication or mental health services…and I respect that that's where they're at and clearly, I mean I would love to see everyone getting better and be able to self-manage if they can, but not everyone will be able to…I have some connection with them, like I can see that they really struggle with the concept, so it is, it's really difficult…I don't want people to go to hospital, but I don't want them getting worse and putting themselves at risk in the community…it's a fine line.*

*(Robyn) I guess I put myself in that situation…’I'm being essentially dragged along to hearing about something I want nothing to do with about medication I don't like and people who just want to interfere in my life.’ So I kind of put myself in that situation from their perspective and so I try and, I guess, use the language that won't upset my clients, because I'm very conscious too that I need to maintain rapport to continue to working…I think empowering them at every opportunity so they have a sense of making decisions for themselves and not having other people make it, so even though there's like a treatment order in place it's about saying, ‘Look, just because there's a treatment order there, it doesn't mean that you can't choose where you go to have your depot or what depot you want to have", and so I try and work with people in that way, because I rather like the recovery model.*

*(Robyn) I think if more people maybe put themselves in the place of the client with what they know about the medication, the illness, and life in general, I mean a CTO would be pointless for me if I was on medication. I'd go interstate. I'd be out of here. No way. And I would be a pain. I would really want to have the least, and I'd want a trial…So understand where some of these clients come from because I'd be exactly the same because it's shit medication with awful side effects, just awful. But the other side of it I think, too, is being within mental health and seeing people who have avoided medication for a long period of time and seeing the cognitive deficits that they actually experience now, that to me is really sad, really sad. And particularly people who, when I first started out, there was a lot of potential for them to do well.*

Some of the patient participants described how they perceived a lack of empathy from their workers:

*(Jessica) The psychiatrist sat at the other end of the room also, and showed no empathy at all for my circumstances. ‘They cut their teeth in the public system; they wield their power and have no compassion at all.*

John talked about the lack of capacity of workers to be in the space he was in when he was acutely psychotic, and mute. It suggested that another way of looking at empathy might be, not as an ability to transport your thinking into the thoughts of somebody else, but about some strategies to make people feel able to start, to give them some time so that they can start to work things through. For John, the people that helped him might not have had a clue what was going on for him.

*(Interviewer) So you do you think that they're not quite sure how to talk to you?*

*(John) Yeah, how to approach me.*

*(Interviewer) Yeah, because they're still trying to work out how to unpack that stuff with you?*

*(John) Exactly.*

No worker participants’ comments suggest that they thought it was impossible to have empathy with someone who is seriously psychotic. Some talked about ways to get some purchase on that space in order to engage patients. There is a growing literature on the importance and ways of delivering empathy training for health care professionals [46]. Some workers appeared to emphasise, through their comments, how hard it is to acquire empathy within the system as it stands. The interviewer perceived that workers appeared to value the opportunity to reflect more deeply on the tensions inherent in working with patients on CTOs; that this was not necessarily something they did in the day-to-day dialogues with peers. Worker participants explained that the system does not support building empathy, but that empathy training would be an important addition to support their delivery of care.

## Discussion

This study revealed many examples of moral framing present in the language used by both worker and patient participants. There were a range of interactions and positions where morality was constructed to evaluate or justify what happened including what led to a CTO being imposed or not, and about experiences during the CTO period. For many worker participants, this involved being benevolent towards patients, acting virtuously towards them, and softening the coercive stick inherent in the CTO process through attempts to empathize with patients’ experience. However, this seemed to be more about the worker wanting people to appear to be better, to be more socially acceptable, rather than the person actually feeling better about whom they are and what they feel or think; so that the worker then felt better about themselves and their effectiveness as a worker. For almost all patient participants, the CTO experience was also understood as a morally framed one, of them being punished for being bad, being seen as untrustworthy, and having faults to be corrected via coercive mental health services practices that worked against their full engagement in the recovery process.

The term ‘coercion’ is largely absent from mental health legislation and other documents used within mental health services. However, coercion was a prominent concern for participants, with workers ever conscious of its potential presence in their actions towards patients, and patients expressing its presence in their interactions with the CTO process. Worker participants used a range of arguments to justify the use of coercion. Some appeared to get caught up in the process of making threats and offers, as part of their interactions with patients. Szmukler and Appelbaum [48] examine in detail the moral distinctions between each form of coercion used in the context of mental health treatment. They identify these as persuasion, interpersonal leverage, inducements, threats, and compulsory treatment. They further explain the difference between threats and offers as their moral baseline: a threat is apparent when the person being made the offer is worse off than their baseline position if they do not accept the offer; it is an offer when the person is no worse off if they do not accept the offer.

Szmukler and Appelbaum [48] further examine the concepts of hard paternalism (actions taken in the patient’s best interests without their consent, where the patient believes they could make their own decision) and soft paternalism (actions taken only if the patient lacks decision-making capacity, where treatment is in their best interests). Rhodes [49] argues that the patient’s perception of what will happen if they do not accept the offer is an important and necessary condition for coercion. Likewise, Dunn et al. [24] argue that, “ threatening to act in a way that would equate with a failure to uphold the requirements of these duties is wrong, irrespective of the benefit accrued through treatment adherence” (p.1). Patient participants in our study described a range of these forms of paternalism. They clearly stated that they wanted to make decisions for themselves. Worker participants’ comments clearly show that many struggled ethically with administering CTOs because they had a strong desire to minimize coercion and paternalism. Fishwick et al. [50] ask, “Can a paternalistic action be justified ethically?” (p.191). Carney [51] warns against ‘hidden coercion’ within calls for the positive right to treatment as a justification for CTOs. Of note, one of our worker participants argued for more first episode psychosis patients to be put on CTOs so that they would receive earlier, more assertive treatment and avoid the long-term negative consequences of untreated mental illness. What such a view fails to appreciate is the damage to trust, worker-patient relationships and engagement with healthcare providers that such a path can bring, as described by many of our patient participants, and noted in other studies [9, 11–14, 35, 36]. O’Hagan refers to coercion as the ‘elephant in the recovery room’ [52] and that, to make progress, we need to have more open dialogue about paternalism and coercion and their association with the moral framings that both workers and patients use to describe their experience of CTOs.

Several workers’ descriptions of their interactions with patients on CTOs appeared to focus on medication compliance as central to their practice. Several patient participants perceived this as the main agenda of workers’ interactions with them, at the exclusion of other forms of support. Stratford et al. [53] acknowledge this focus on medication as one of a number of challenges facing mental health care in Australia, impeding the growth of recovery-oriented approaches. They argue that this focus deskills workers and encourages coercion as a currency for engaging with and treating people with mental illness, and that it leads to disappointment for patients and their families when medication is viewed as the solution to treatment regardless of significant medication side effects.

Current Australian mental health legislation appears to focus on the process of imposing CTOs, with little accountability for what workers, services and patients do during the CTO period. This is of particular concern when workers seek to further the period of a CTO beyond its initial imposition. To address this concern, recent revisions of the Mental Health Act within Australian state jurisdictions have involved greater emphasis on requiring workers to clearly demonstrate their decision-making processes when seeking to impose a CTO. This, in-turn, can help drive cultural reform of mental health services because it frames the culture and moral values within which people are working. However, legislation also runs the risk of being a blunt instrument when translated into practice. For example, in Victoria in the 1980s, and more recently in South Australia, treatment and care plan requirements within the legislation, despite the good intention to ensure greater accountability of care, have continued to largely focus on medication. Ultimately, as Brophy recently argued, ‘You cannot legislate for compassion and respect’ ([54], see also [55]).

Fishwich et al. [50] emphasize that the concept of the therapeutic relationship is fundamental to mental health care, despite inherent tensions between the caring imperative and custodial functions embodied in mental health legislation. This is further hampered by dialogues about risk and dangerousness which work against reform because they give power to those assessing risk without constraints, which then distort mental health workers’ and the community’s cultural values about people with mental health issues. Several participants in our study confirmed these concerns. The Critical Psychiatry Movement [56] has argued that the non-technical aspects of interventions (such as the development of meaningful, non-judgmental relationships) are as much involved in recovery as the therapies and psychiatric medications used to treat mental illness. They appear to be describing a need for greater empathy for patients’ experience. Other researchers have found that the relationship between workers and patients is an important factor that can either assist or obstruct patients’ recovery [14, 57–59].

Denhov and Topor’s [60] study involving qualitative interviews with 71 patients in Sweden revealed the importance that patients placed on their experiences of treatment and care, from a moral values perspective. Perception of professionals’ underlying attitude toward them, and trusting the health professional, were significant concerns, were central to the relationship, and took time. The emotional climate in the relationship was also pivotal to whether it was perceived by patients as helpful or not. Their participants described particular characteristics of helpful professionals: “nice, friendly, humane, attentive, obliging, helpful, patient, genuinely interested and genuinely involved” (p.420). The assumption made by our worker participants was that patients should trust them, like them because they have good intentions, and engage with them because they are there to help.

Our results illustrate the important but difficult role of empathy when engaging people on CTOs and they corroborate points made in the literature about the need for workers to receive ongoing empathy training. With processes focused on risk management, workers are inadvertently forced into focusing on symptoms and compliance: ‘Have you behaved? Have you been good?’ Whereas, “Helping professionals seem able to convey that they regard the patient as an ordinary human being who is something more than merely a patient” (p.420). [60] Light et al. [37] offer a number of solutions to alleviate distress arising from being on a CTO. These include: clearer communication about the CTO between workers, patients and carers; improved access to mental and physical health services; and, acknowledgement that distress is an inherent part of the CTO experience for people with severe mental illness. The first and third of these suggestions likely require workers to develop greater empathy for their patients. Want and Wand [28] argue broadly for more comprehensive education about mental health legislation for mental health professionals. Our results suggest a need to create opportunities for workers to self-reflect and receive constructive feedback from others. Banks and Gallagher [61] emphasize that the virtuous practitioner is about the character and ‘being’ of the worker, not just their conduct or actions.

For several patient and worker participants, the patients’ views were no longer seen as valid or truthful; as Vicky and Peter exemplified, ‘There was no second chance at trust’. This meant that shared commitment to reaching agreement was difficult and workers were more likely to use coercive practices which then undermined cooperation [13]. Some workers in our study said, ‘When does the person get the chance to prove that they can do this on their own?’

### Limitations

This study involved a small sample of patients and workers. All patient participants were currently on a CTO which might have influenced their perceptions, due to potentially varying levels of understanding of their mental health. Despite this, our priority was to elicit their perspective of their experiences of CTOs and many described feeling of coercion in detail. Soininen et al’s [62] systematic review of research on the challenges in studying patients’ perspectives of coercion was only applied to studies involving inpatients. A similar study could be undertaken with community samples. All worker participants were drawn from one mental health service in Australia. Therefore, results may not be generalizable to other jurisdictions. Other studies have noted bias in recruiting participants who might have more positive regard for CTOs [37]. Our study did not have this limitation. The interviewer’s status as a consumer advocate may have assisted participants to speak more freely.

The lead researcher was a clinician within mental health services between 1996 and 2007. She was aware of 3 of the patient participants because they were clients of one of the services where she worked; but she had not been directly involved in their care as a case manager at any time.

The lead researcher was aware of 6 of the staff participants because of her prior clinical role within mental health services. She had not had any direct or regular contact with any of these participants since leaving mental health services in 2007, nor any ongoing connection to any of the participants in this study. All participants were unknown to all other members of the research team.

## Conclusions

More than ever, critical reflection on our assumptions and values about mental illness is needed for the dialogue of recovery to happen. Experiences of CTOs are multi-layered, varied and depend critically upon empathy and self-reflection of patients and workers; on the relationship between what is done and how it is done. Robust ethical debate is needed in addition to more empirical evidence for the effectiveness of CTOs and other modes of engaging these patients in care [8]. This includes explicit examination and reflection on the moral framings present in the everyday work and interactions between mental health workers and their patients, in order to overcome the paradox of the moral grey zone between caring and controlling.

## Abbreviations

CTO: Community treatment order
